# Mitogenomic Comparison of the Mole Crickets Gryllotalpidae with the Phylogenetic Implications (Orthoptera: Ensifera)

**DOI:** 10.3390/insects13100919

**Published:** 2022-10-11

**Authors:** Yan Ma, Ying Miao

**Affiliations:** College of Agriculture, Ningxia University, Yinchuan 750021, China

**Keywords:** Gryllidea, *Gryllotalpa*, mitochondrial genome, phylogeny

## Abstract

**Simple Summary:**

We sequenced the complete mitochondrial genomes (mitogenomes) of *Gryllotalpa henana* Cai & Niu, 1998 and the Chinese *G. orientalis* Burmeister, 1838 for the first time, and reconstructed the mitogenomic phylogeny of the infraorder Gryllidea. The results show that the two new mitogenomes are double-stranded circular molecules with a typical gene complement, gene arrangement and base composition, the same as those of other gryllotalpids and ancestral insects. Tandem repeats of the control region were discovered in Gryllotalpidae for the first time. Considering both the high nucleotide divergence and the elevated ratio of Ka/Ks, the genes *nad2* and *nad6* may be evaluated as potential markers for future phylogeny and species delimitation in Gryllotalpidae. The results of phylogenetic analyses provide supports for the mitogenomic and transcriptomic trees, but partially contradict those of the multilocus phylogenies.

**Abstract:**

Owing to limited molecular data, the phylogenetic position of the family Gryllotalpidae is still controversial in the infraorder Gryllidea. Mitochondrial genome (mitogenome) plays a crucial role in reconstructing phylogenetic relationships and revealing the molecular evolution of insects. However, only four mitogenomes have been reported in Gryllotalpidae to date. Herein, we obtained the first mitogenomes of *Gryllotalpa henana* Cai & Niu, 1998 and the Chinese *G. orientalis* Burmeister, 1838, made a detailed comparison of all mitogenomes available in Gryllotalpidae and reconstructed the phylogeny of Gryllidea based on mitogenomes using Bayesian inference (BI) and maximum likelihood (ML) methods. The results show that the complete mitogenome sequences of *G. henana* (15,504 bp) and *G. orientalis* (15,497 bp) are conserved, both exhibiting the double-stranded circular structure, typical gene content and the ancestral insect gene arrangement. The complete mitogenome of *G.* *henana* exhibits the lowest average AT content ever detected in Gryllotalpidae, and even Gryllidea. The gene *nad2* of both species has atypical initiation codon GTG. All tRNAs exhibit typical clover-leaf structure, except for *trnS1* lacking the dihydrouridine (DHU) arm. A potential stem–loop structure, containing a (T)_n_(TC)_2_(T)_n_ sequence, is detected in the control region of all gryllotalpids investigated and is likely related to the replication initiation of the minority strand. The phylogenetic analyses recover the six families of Gryllidea as Gryllotalpidae + (Myrmecophilidae + (Mogoplistidae + (Trigonidiidae + (Phalangopsidae + Gryllidae)))), similar to the trees based on transcriptomic and mitogenomic data. However, the trees are slightly different from the multilocus phylogenies, which show the sister-group relationship of Gryllotalpidae and Myrmecophilidae. The contradictions between mitogenomic and multilocus trees are briefly discussed.

## 1. Introduction

The mitochondrial genomes (or mitogenomes) of insects are double-stranded circular molecules with lengths ranging from approximately 15 kb to 20 kb, and generally comprise 37 genes with 13 protein-coding genes (PCGs), 22 transfer RNA genes (tRNAs), two ribosome RNA genes (rRNAs) and a non-coding control region (CR) [1]. Mitogenomes are one of the most information-rich characteristics, and are useful in phylogeny, evolutionary history, species delimitation and population genetics [2,3,4,5]. Such studies have been well documented in many insect groups, and greatly contributed to understanding their phylogeny and evolution [6,7,8,9]. In Gryllotalpidae, however, only four species have available mitogenomes in GenBank to date [10,11,12,13].

Gryllotalpidae is a small family of mole crickets and currently consists of more than 100 species in eight extant genera worldwide [14,15]. Gryllotalpids comprise an exclusive group that possesses a pair of digging forelegs, a tumescent pronotum, short antennae and hind legs lacking jumping ability [16]. Owing to the limited molecular data, the phylogenetic position of Gryllotalpidae in Gryllidea is still controversial. Gryllotalpidae is sister to Myrmecophilidae based on multilocus analysis [17], but has conflicting phylogenetic positions in mitogenome-based trees [8,18].

*Gryllotalpa* Latreille, 1802, characterized by forelegs with four tibial dactyls, is the largest and most widespread genus in Gryllotalpidae and comprises more than ½ the species of the family recorded from all zoogeographical regions, with only 11 species distributed in China [19,20]. The species of *Gryllotalpa* are similar in external morphology, but exhibit complicated variations intraspecifically in morphology of wing venation and male genitalia, leading to difficulties in species delimitation [21,22]. The application of additional characteristics is necessary to resolve the taxonomic problem.

In this study, we present the first complete mitogenomes of *G. henana* and the Chinese *G.* *orientalis*, make a detailed comparison of gryllotalpid mitogenomes, and reconstruct the phylogeny of the infraorder Gryllidea, in an attempt to contribute the mitogenomic data of Gryllotalpidae for future phylogenetic studies of Orthoptera.

## 2. Materials and Methods

### 2.1. Sample Collection and Processing

An adult female of *G. henana* and an adult male of *G. orientalis* were collected at the Danjiang River Beach (33°5′ N, 111°13′ E, elevation 220–240 m) in Xichuan County, Henan Province, China, from late May to middle June 2021. The middle leg on one side of each specimen was stored in dry ice and sent to Biomarker Technologies, Inc. (Beijing, China) for extraction and sequencing. The complete mitogenome sequences were generated using the Illumina HiSeq™ 4000 system. The rest of the specimens were preserved in 75% ethanol and placed in the Laboratory of Agricultural Entomology and Pest Control, College of Agriculture, Ningxia University.

### 2.2. Sequence Analyses

The mitochondrial invertebrate genetic code was selected as the general code for all the programs used in the present study. The raw paired reads were retrieved and quality trimmed by CLC Genomics Workbench v7.0.4 (CLC Bio, Aarhus, Denmark) with default parameters, using the mitogenomic sequence of *G. unispina* (KC894752) and *G. orientalis* (AY660929) as references, respectively. The mitochondrial genomes of *G. henana* and the Chinese *G. orientalis* were annotated with Geneious 8.1.3 [23] with the same references. All 13 PCGs were determined by comparing with the ORF Finder and the homologous sequences of reference mitogenomes. Twenty-two tRNAs and two rRNAs were identified using the MITOS Web Server (http://mitos2.bioinf.uni-leipzig.de/index.py, accessed on 20 June 2022) [24]. Transfer RNAs were manually plotted, according to the secondary structure predicted by MITOS, using Adobe Illustrator CS5 (Adobe Inc., San Jose, CA, USA). Tandem Repeats Finder server (https://tandem.bu.edu/trf/trf.html, accessed on 17 May 2022) [25] and Mfold Web Server (http://www.mfold.org/, accessed on 17 May 2022) [26] were used to identify tandem repeats and to infer the stem-loop structure, respectively. Mitogenome maps were drawn using OGDRAW [27].

The base composition, codon usage and relative synonymous codon usage (RSCU) were all calculated using PhyloSuite [28]. DnaSP 6.0 [29] was used to conduct the nucleotide diversity (Pi), and non-synonymous (Ka) and synonymous (Ks) substitutions of each PCG among the species of Gryllotalpidae. Sliding window analyses with a window of 100 bp and a step size of 25 bp were performed to estimate the sequence diversity for each independent PCG, using DnaSP 6.0. Genetic distances based on 13 PCGs were estimated using MEGA 7.0 with Kimura-2-parameter (K2P) [30]. AT-content (the proportion of A + T out of the total) was used to assess the overall composition of the double-stranded molecule [31]. Strand asymmetry was calculated according to the formula: AT-skew = (A − T)/(A + T) and GC-skew = (G − C)/(G + C) [32]. The AT-content, AT-skew and GC-skew were graphically plotted by Origin 2018 (OriginLab Corp., Northampton, MA, USA). The Pi values were graphically plotted by CorelDRAW 2020 (Corel Corp., Ottawa, ON, Canada). The genetic distance and Ka/Ks ratios were graphically plotted by Microsoft Excel spreadsheet.

### 2.3. Phylogenetic Analyses

Twenty-eight species from six families of Gryllidea were chosen as the ingroup, and four species in Tettigoniidea and one species in Schizodactyloidea were selected as outgroups. The detailed information of species used in phylogenetic analyses were listed in Table 1. Statistics for the basic characteristics of the mitogenome and the extraction of PCGs and rRNAs were produced by PhyloSuite. The alignment of all 13 PCGs was conducted in batches with MAFFT integrated into PhyloSuite with codon alignment mode setting [33,34]. Two rRNAs were aligned using the Q-INS-i algorithm incorporated into MAFFT-with-extensions software (http://mafft.cbrc.jp/alignment/server/, accessed on 29 March 2022) [33]. Ambiguous sites of alignments of all genes were manually removed, and the modified alignments were concatenated using PhyloSuite [34].

Phylogenetic analyses were conducted using four different datasets: (1) P123: 13 PCGs (10,899 bp), (2) P123R: 13 PCGs + 2 rRNAs (13,550 bp), (3) P12: 13 PCGs excluding the third codon position (7266 bp), (4) P12R: 13 PCGs excluding the third codon position + 2 rRNAs (9917 bp). Phylogenetic trees were reconstructed using Bayesian inference (BI) and maximum likelihood (ML) analyses, with partition strategies for analyzing mitogenome data according to Leavitt [35]. The best-fit partition schemes and models for BI analyses were inferred using PartitionFinder 2 [36] integrated into PhyloSuite [34], and are shown in Supplementary Table S1. BI trees were conducted using MrBayes 3.2.6 [37] with 10 million MCMC generations, sampling every 1000 generations. The convergence was considered to be reached when the average standard deviation of the split frequencies was lower than 0.01. The first 25% were discarded as “burn-in”, and the remaining samples were used to generate the majority consensus trees and estimate the posterior probabilities (PPs). The best-fit substitution models for ML analyses were selected by ModelFinder [38], and shown in Supplementary Table S2. ML trees were reconstructed using IQ-TREE integrated into PhyloSuite under Ultrafast bootstrap. Bootstrap supports (BSs) were evaluated with 1000 replicates.

**Table 1:** Details of the species investigated and the relative information.

Superfamily	Family	Species	Locality	Size (bp)	Accession No.	Resource
Gryllotalpoidea	Gryllotalpidae	*Gryllotalpa henana* Cai & Niu, 1998	China	15,504	ON243749	This study
		*G. orientalis* Burmeister, 1838	China	15,497	ON210982	This study
		*G. orientalis* Burmeister, 1838	Korea	15,521	AY660929	[12]
		*G. pluvialis* (Mjöberg, 1913)	Australia	15,525	EU938371	[11]
		*Gryllotalpa* sp.	China	15,506	MK903562	[10]
		*G. unispina* Saussure, 1874	China	15,513	KC894752	[13]
	Myrmecophilidae	*Myrmecophilus kubotai* Maruyama, 2004	Japan	15,345	MZ440658	[18]
		*M. manni* Schimmer, 1911	USA	15,323	EU938370	[11]
		*Myrmecophilus* sp.	Japan	15,341	MZ440659	[18]
Grylloidea	Phalangopsidae	*Meloimorpha japonica* (Haan, 1844)	China	15,880	MH580273	[39]
		*Cacoplistes rogenhoferi* Saussure, 1877	China	16,018	MH580272	[39]
	Mogoplistidae	*Ornebius bimaculatus* (Shiraki, 1930)	China	16,136	MH580274	[39]
		*O. fuscicerci* (Shiraki, 1930)	China	16,368	MH580275	[39]
		*O. kanetataki* (Matsumura, 1904)	China	16,589	MH580276	[39]
	Trigonidiidae	*Dianemobius fascipes* (Walker, 1869)	China	15,363	MK303550	[40]
		*D. furumagiensis* (Ohmachi & Furukawa, 1929)	China	15,350	MK303551	[40]
		*Homoeoxipha nigripes* Xia & Liu, 1993	China	15,679	MK303553	[40]
		*Natula pravdini* (Gorochov, 1985)	China	15,817	MG701239	[40]
		*Svistella anhuiensis* He, Li & Liu, 2009	China	16,494	MG701238	[40]
		*Polionemobius taprobanensis* (Walker, 1869)	China	16,641	MK303552	[40]
	Gryllidae	*Gryllodes sigillatus* (Walker, 1869)	China	16,369	MW365703	[41]
		*Gryllus bimaculatus* De Geer, 1773	Korea	16,075	MT993975	[42]
		*Loxoblemmus doenitzi* Stein, 1881	-	15,620	KX673202	[43]
		*Oecanthus sinensis* Walker, 1869	China	16,142	KY783908	[44]
		*Truljalia hibinonis* (Matsumura, 1917)	China	15,120	KY783909	[44]
		*Turanogryllus eous* Bey-Bienko, 1956	China	16,045	MK656322	[45]
		*Velarifictorus hemelytrus* (Saussure, 1877)	China	16,123	KU562918	[46]
		*Acheta domesticus* (Linnaeus, 1758)	Japan	16,071	MZ440654	[18]
Outgroup	Tettigoniidae	*Alloxiphidiopsis emarginata* (Tinkham, 1944)	China	16,207	MN562488	[47]
		*Tettigonia chinensis* Willemse, 1933	-	16,244	KX057727	[48]
	Gryllacrididae	*Camptonotus carolinensis* (Gerstaecker, 1860)	-	15,211	KM657333	[49]
	Schizodactylidae	*Comicus campestris* Irish, 1986	-	15,691	KM657337	[49]
	Prophalangopsidae	*Tarragoilus diuturnus* Gorochov, 2001	China	16,144	JQ999995	[50]

## 3. Results and Discussion

### 3.1. Genome Structure and Base Composition

**Figure 1:**
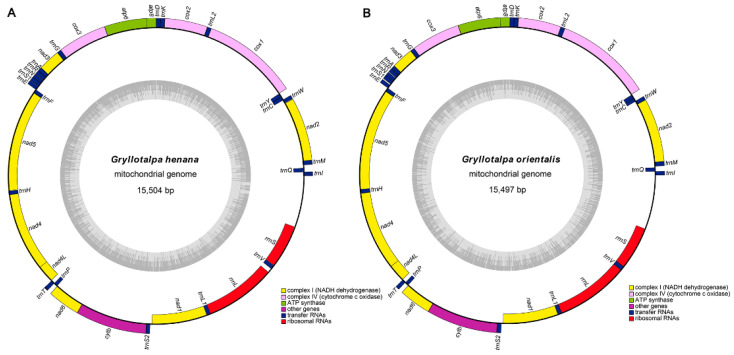
Mitochondrial genome arrangements. (**A**) Mitochondrial genome of *Gryllotalpa henana.* (**B**) Mitochondrial genome of *Gryllotalpa orientalis*. The J-strand is visualized on the outer circle and the N-strand on the inner circle. The dark and light areas of the grey inner circle represent the GC- and AT-content, respectively.

The complete mitogenomes of *G. henana* (Figure 1A) and the Chinese *G. orientalis* (Figure 1B) are 15,504 bp and 15,497 bp representing the smallest sizes known in Gryllotalpidae (Table 1). The lengths of two new mitogenomes are quite conserved, and within the size range of orthopteran mitogenomes (14–17 kb) [35,48,51]. Size differences of the mitogenomes in Gryllotalpidae are mainly due to variations in the length of the control region (CR) and the intergenic spaces between some of the tRNAs [10,11,12,13]. The mitogenomes of both species, similar to those of other gryllotalpids, are circular double-stranded molecules and contain the complete set of 37 genes (13 PCGs, 22 tRNAs and two rRNAs) and a non-coding CR (AT-rich region) (Figure 1). The majority strand (J-strand) encodes 23 genes, including *nad2*, *nad3*, *nad6*, *cytb*, *cox1*, *cox2*, *cox3*, *atp8*, *atp6*, *trnI*, *trnM*, *trnW*, *trnL2*, *trnK*, *trnD*, *trnG*, *trnA*, *trnR*, *trnN*, *trnS1*, *trnE*, *trnT* and *trnS2*. The remaining 14 genes (*nad1*, *nad4*, *nad4L*, *nad5*, *trnQ*, *trnC*, *trnY*, *trnF*, *trnH*, *trnP*, *trnL1*, *trnV*, *rrnL* and *rrnS*) are encoded on the minority strand (N-strand). The mitogenomes obtained herein are identical to those of other gryllotalpids in gene order and gene orientation, which are the hypothesized ancestral arrangements found in several insect orders [1].

**Figure 2:**
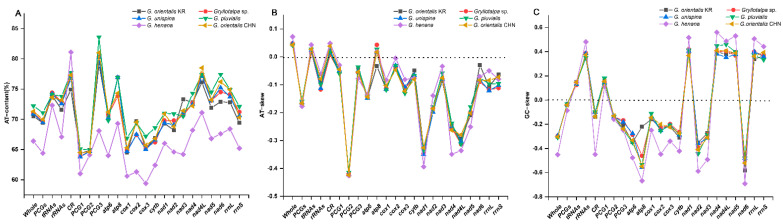
Comparison of AT content and nucleotide skewness of six species in Gryllotalpidae. (**A**) AT-content. (**B**) AT-skew. (**C**) GC-skew. CHN, China; KR, Korea.

Two separated features, base proportion (AT-content) and strand asymmetry (AT- and GC-skew), are used to assess the base compositional bias of mitogenomes [31,32]. The AT-content of the whole mitogenomes ranges from 66.4% in *G. henana* to 72.2% in *G. pluvialis* (Figure 2A, Table 2), indicating that the overall composition is biased towards A and T in Gryllotalpidae. The CR in all six species exhibits a higher value of the AT-content (74.9–81.1%), followed successively by tRNAs (72.3–74.4%), rRNAs (67.1–73.8%) and PCGs (64.4–71.1%). In PCGs, the third codon positions have the highest AT-content (68.1–83.6%), compared with the first (61.0–65.1%) and second codon positions (64.1–64.9%) in all gryllotalpids. The AT-contents of four genomic regions are generally lower in *G. henana* than in other five gryllotalpids (Figure 2A, Table 2). The entire mitogenomes of all gryllotalpids exhibit the typical skew pattern of insects with positive AT-skew (0.042–0.072) and negative GC-skew (−0.451–−0.295), indicating that the majority strand of mitogenomes is biased in favor of A and C (Figure 2B,C, Table 2). The skew patterns of the four genomic regions are conserved in *Gryllotalpa*, and exhibit negative AT- and GC-skew in PCGs, positive AT- and GC-skew in tRNAs, negative AT-skew and positive GC-skew in rRNAs, and positive AT-skew and negative GC-skew in CR. The values of AT-skew are small and not significantly different from zero in the four genomic regions except for the PCGs (−0.177–−0.159). The GC-skew values are also low, with the exception of the increased ones for rRNAs in all the species of *Gryllotalpa* (0.347–0.482) and the decreased one for CR in *G. henana* (−0.450).

### 3.2. Protein-Coding Genes and Codon Usage

The concatenated sequence of the PCGs is 11,097 bp in *G. henana* and 11,109 bp in the Chinese *G. orientalis*, accounting for 71.6% and 71.7% of their whole mitogenomes, respectively (Table 2 and Table 3). The 13 PCGs of the two new mitogenomes, similar to those of the other four gryllotalpids, contain two ATPase subunits (*atp6* and *atp8*), three cytochrome c oxidase subunits (*cox1*–*3*), one cytochrome b gene (*cytb*), and seven NADH dehydrogenase subunits (*nad1*–*6* and *nad4L*) (Figure 1, Table 3). The lengths of the 13 PCGs range from 156 bp of *atp8* to 1723 bp of *nad5* in both mitogenomes newly sequenced. The shortest *atp8* and longest *nad5*, also found in other four gryllotalpids, are common features in metazoan mitogenomes [52,53].

**Figure 3:**
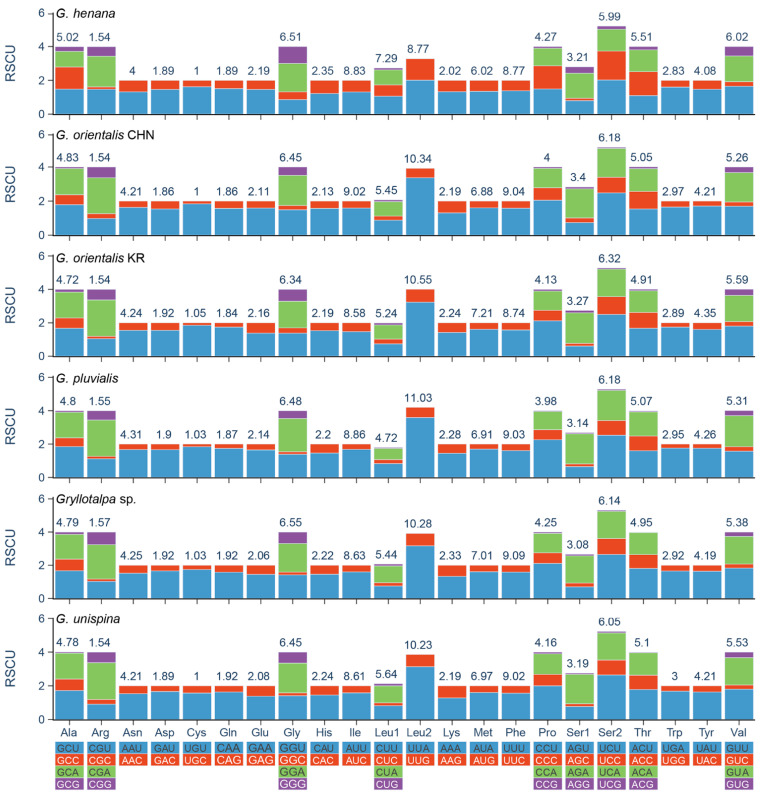
Relative synonymous codon usage (RSCU) of the mitochondrial genomes of six species in Gryllotalpidae. CHN, China; KR, Korea. The numbers above the colored columns indicate the frequencies of amino acids.

All PCGs of the two new mitogenomes have the typical initiation codon (ATN), except for *nad2* starting with GTG (Table 3). The atypical initiation codon is also present in the mitogenomes of two other mole crickets (the Korean *G. orientalis* and *G. unispina*) and two katydids (*Kuwayamaea brachyptera* Gorochov & Kang, 2002 and *Ruidocollaris obscura* Liu & Jin, 1999) [12,13,54]. The termination codons are relatively conserved in Gryllotalpidae. Most of them are complete triplet bases TAA/TAG, and others are incomplete T/TA immediately followed by or partially overlapped with a tRNA gene. Incomplete stop codons are fairly common in the orthopteran mitogenomes and can be converted into a potential stop codon via polyadenylation to TAA [18,39,40,55]. The results of RSCU analyses show that the PCGs exhibit strong biases toward the nucleotides A and U in the codon usage. The four most frequent codons (UUU/Phe, UUA/Leu2, AUU/Ile, and AUA/Met) are the same in Gryllotalpidae, and all composed wholly of A or U (Figure 3, Supplementary Table S3). The codons ending with A/U occur more frequently than that with G/C, suggesting that the AU composition at third position of codons positively influences the nucleotide AT (or AU) bias of the PCGs in Gryllotalpidae.

### 3.3. Transfer and Ribosomal RNA Genes

**Figure 4:**
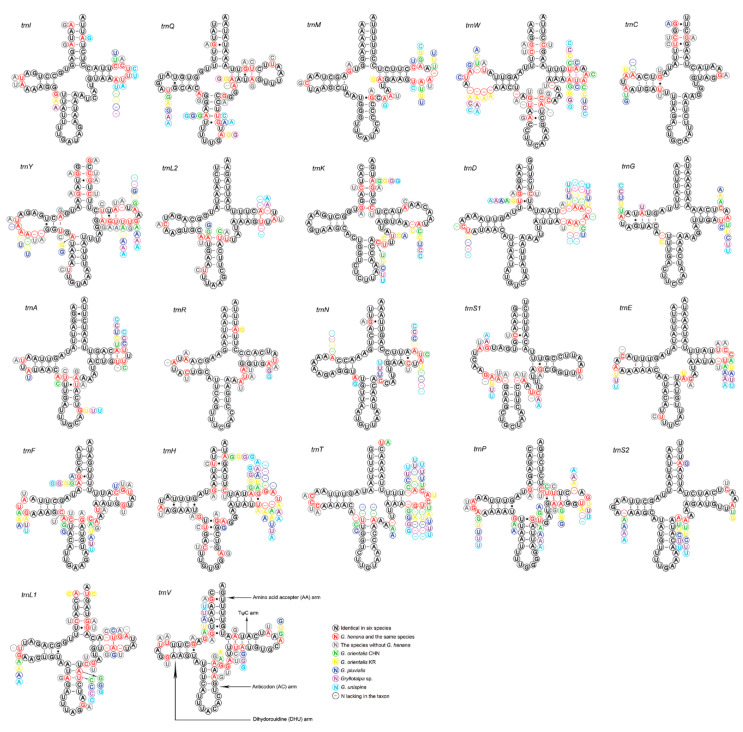
Secondary structure for the tRNAs of six species in Gryllotalpidae. CHN, China; KR, Korea.

The 22 tRNAs of the two new mitogenomes are scattered around the circular DNA molecule, and are arranged identically in order and direction (Figure 1). The tRNAs of gryllotalpids retain the ancestral gene order [10,11,12,13], whereas multiple patterns of tRNA rearrangements have been detected in many other ensiferans [39,48] and most caeliferans [35]. All tRNAs exhibit typical clover–leaf structure, except for *trnS1* (Figure 4). The dihydrouridine (DHU) arm of *trnS1* forms a simple loop as in many other metazoans including gryllotalpids [10,11,12,13,52,56]. The length of tRNAs varies from 62 bp (*trnC*) to 71 bp (*trnK*) in *G. henana* and from 61 bp (*trnC*) to 71 bp (*trnK*) in the Chinese *G. orientalis* (Table 2), both within the variation range in Gryllotalpidae. The *trnG* gene of gryllotalpids generally exhibits the lowest nucleotide substitutions, while *trnL1*, *trnW* and *trnY* genes tend to be more variable among 22 tRNA genes (Figure 4). All tRNAs in the mitogenomes of Gryllotalpidae possess invariable length of 7 bp for both the acceptor stem and the anticodon loop. The length of anticodon stem is relatively conservative, varying from 4 bp in *trnK* and *trnM* to 5 bp in the rest of tRNAs. Most of the size variations among tRNAs stemmed from the length variation in DHU and TψC arms, within which the size of loops (all 3–10 bp) is more variable than that of stems (all 3–5 bp).

The tRNAs of *G. henana* possess a total of 36 unmatched base pairs, including 31 GU mismatches in most tRNAs, three AC mismatches in the anticodon stem of *trnS1* and *trnW* and the TψC stem of *trnN*, and two UU mismatches in the acceptor stem of *trnD* and the anticodon stem of *trnA* (Figure 4, Supplementary Table S4). A total of 30 mismatches were detected in the Chinese *G. orientalis*. Twenty-seven of them are GU pairs, two are UU mismatches in the DHU stem of *trnC* and the acceptor stem of *trnL1*, and one is AA pair in the anticodon arm of *trnS1*. The mismatch number in the Chinese *G. orientalis* is lower than that in the Korean one (36 mismatches) (Supplementary Table S4), suggesting that the mitogenomes are differentiated intraspecifically.

The two rRNA genes (*rrnL* and *rrnS*) are located in the conserved positions as in mitogenomes of other gryllotalpids [10,11,12,13]. *rrnL* is present between *trnL1* and *trnV*, while *rrnS* between *trnV* and the CR (Figure 1). The two genes *rrnL* and *rrnS* are 1214 and 733 bp long in *G. henana*, and 1236 and 730 bp long in the Chinese *G. orientalis*, respectively (Table 3). The lengths range from 1214 to 1247 bp for *rrnL*, and from 719 to 783 bp for *rrnS* in Gryllotalpidae. The AT content of rRNAs is 67.1% in *G. henana* and 73.2% in the Chinese *G. orientalis*. The value of AT content is lower in *G. henana* than those in the other gryllotalpids and many other orthopterans [12,48,57,58,59,60,61,62,63].

### 3.4. Intergenic Spacers and Gene Overlaps

In *G. henana*, intergenic spacers are distributed in 12 regions and range in size from 1 to 71 bp with a total of 162 bp (Table 3). Eleven intergenic spacers exist in the mitogenome of the Chinese *G. orientalis*, ranging from 1 to 25 bp and adding up to 73 bp. The largest has 71 bp located between *rrnL* and *trnV* in *G. henana*, whereas there are 25 bp located between *trnS2* and *nad1* in the Chinese *G. orientalis*. Two identical intergenic spacers were detected in the mitogenomes of all gryllotalpids. One is between *nad4L* and *trnT* (2 bp), and the other is between *nad1* and *trnL1* (1 bp). In most cases, the intergenic spacers consist of only 1 or 2 bp.

The gene overlaps of *G. henana* are distributed in 13 locations with a total of 52 bp, whereas those of the Chinese *G. orientalis* are in 10 locations with a total of 34 bp (Table 3). The longest gene overlap is 17 bp between *trnL1* and *rrnL* in *G. henana*, and 8 bp between *trnW* and *trnC* in the Chinese *G. orientalis*. All six gryllotalpids have five identical overlapping regions, including *trnK*-*trnD* (1 bp), *trnE*-*trnF* (2 bp), *trnI*-*trnQ* (3 bp), *nad4*-*nad4L* (7 bp) and *trnW*-*trnC* (8 bp). In general, the variability of gene overlaps is lower than that of intergenic spacers.

### 3.5. Control Region

The CR, also called AT-rich region, is located in the conserved position between *rrnS* and *trnI* (Figure 1, Table 2). The AT-content of this region is 81.1% in *G. henana* and 77.3% in the Chinese *G. orientalis*. In all six gryllotalpids, the Korean *G. orientalis* shows the lowest AT content of 74.9%, whereas *G. henana* exhibits the highest 81.1%. The CRs of Gryllotalpidae show low variations in lengths, which range from 863 bp in *G. henana* to 920 bp in the Korean *G. orientalis*. The low variations of CR in length are likely attributed to the lacking of conspicuous repeats, which are often found in other insects [6,54,64,65,66]. Two kinds of short repeats were detected in Gryllotalpidae for the first time (Supplementary Table S5). One is the microsatellite (TA)_n_ element found in *G. henana* and the Chinese *G. orientalis*. The other recognized in *G. pluvalis* is the duplicated tandem repeat, containing 18 bp (ATATAATTAAATATTTAA) with 2.3 copies. A potential stem–loop structure, containing (T)_n_(TC)_2_(T)_n_ sequences, was detected in the CR near the *trnI* gene of *G. henana* and the Chinese *G. orientalis*, same as the findings in other gryllotalpids (Figure S1). Similar structures were also found in many crickets of Gryllidea [46,67], and likely related to replication initiation of the N-strand [68].

### 3.6. Genetic Diversity and Selective Constraints

**Figure 5:**
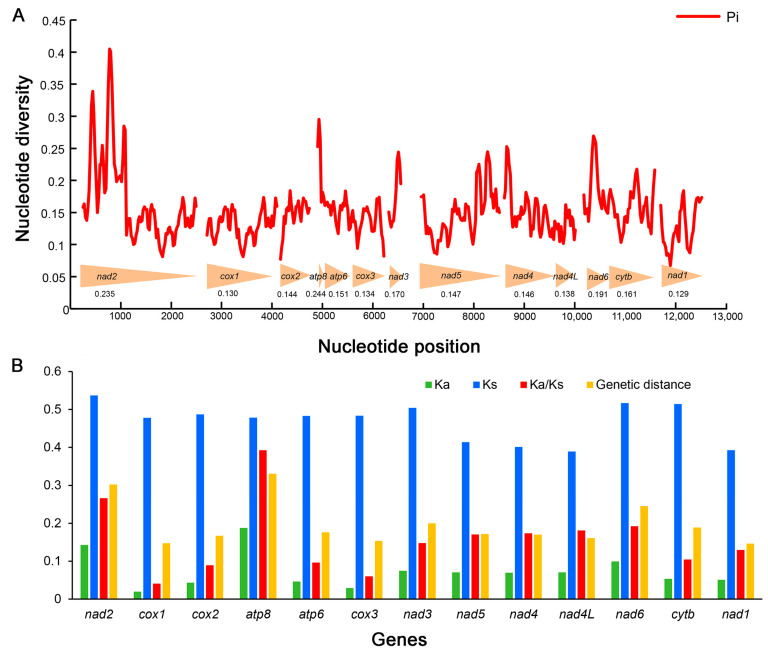
Genetic diversity and selection pressure among 13 protein-coding genes (PCGs) in Gryllotalpidae. (**A**) Sliding window analyses with a window of 100 bp and a step size of 25 bp for 13 PCGs. The red curve shows the values of nucleotide diversity (Pi). The arrowheads at the bottom illustrates the position of each PCG. The Pi value of each PCG is shown below the arrowheads. (**B**) Genetic distance and Ka/Ks ratio of each PCG in Gryllotalpidae. Ka, non-synonymous substitution rates; Ks, synonymous substitution rates.

Sliding window analyses exhibit the estimations of nucleotide diversity (Pi) for each PCG of the six mitogenomes (Figure 5A, Supplementary Table S6). The gene *atp8* has the highest nucleotide diversity (Pi = 0.244), followed by *nad2* (Pi = 0.235) and *nad6* (Pi = 0.191). The genes *cox3* (Pi = 0.134), *cox1* (Pi = 0.130) and *nad1* (Pi = 0.129) are the lower variable. A similar pattern was also detected in terms of mean genetic distances (Figure 5B). *atp8*, *nad2* and *nad6* show high distances with 0.331, 0.302 and 0.245, whereas *cox3*, *cox1* and *nad1* exhibit low distances with 0.154, 0.147 and 0.146, respectively.

Ka/Ks ratio (ω) is an important indicator for detecting molecular adaptation correlated to the biological evolution [69,70]. The Ka/Ks ratios of 13 PCGs are all lower than 1 in all mitogenomes of Gryllotalpidae (Figure 5B, Supplementary Table S6), indicating that these PCGs are evolving under purifying selection and suitable for phylogenetic reconstructions in Gryllotalpidae. The Ka/Ks of *atp8* (ω = 0.393), *nad2* (ω = 0.266) and *nad6* (ω = 0.192) are much higher than those of other PCGs, suggesting that the former three genes experience more relaxed evolutionary constraints and retain more non-synonymous mutations. The gene *cox1* exhibits the lowest Ka/Ks ratio (ω = 0.041) implying the greatest evolutionary limitation on *cox1* among 13 PCG genes. The strong evolutionary constraints (ω << 1) of mitochondrial PCGs suggest that the deleterious mutations are eliminated by purifying selection to maintain highly conserved genes that encode core subunits of the respiratory chain complexes [10,71].

The species of Gryllotalpidae are similar in external morphology but exhibit complicated variations in genitalia, leading to taxonomic difficulties based solely on morphological characters [21,22]. Designing species-specific markers is crucial for resolving such problems. The *cox1* gene has long been used as a universal DNA marker for species identification in insects [72,73,74,75], but is the most conservative protein coding gene in mitogenomes of gryllotalpids. Considering both the high nucleotide divergence and the elevated ratio of Ka/Ks, the genes *nad2* and *nad6* may be evaluated as potential markers for species delimitation in Gryllotalpidae.

### 3.7. Phylogenetic Analyses

**Figure 6:**
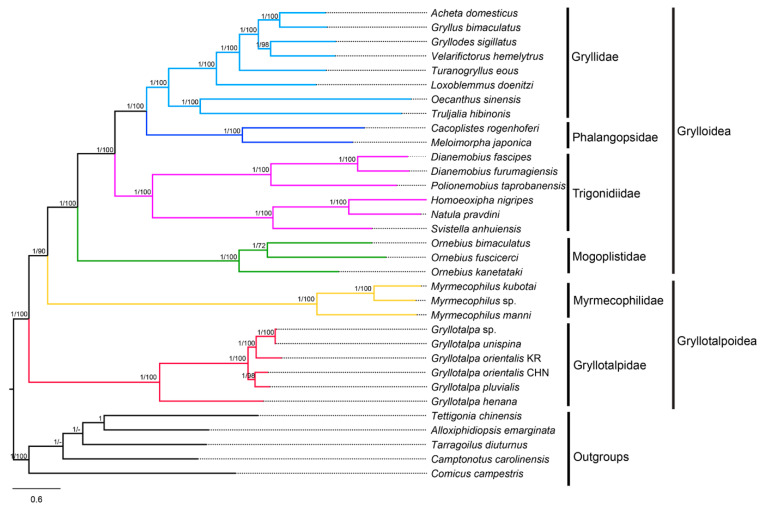
Phylogenetic tree produced by Bayesian inference (BI) based on the P123 dataset. Numerals at nodes are Bayesian posterior probabilities (PPs) and bootstrap support values (BSs), respectively. “-” indicates that the positions of *Tarragoilus diuturnus* and *Camptonotus carolinensis* in the maximum likelihood (ML) tree are slightly different from those in the BI tree.

The phylogenetic trees based on the four datasets (P12, P12R, P123 and P123R) are highly consistent, except for the positions of the Korean *G. orientalis* and *Velarifictorus hemelytrus* (Saussure, 1877) (Figure 6, Supplementary Figures S2–S4). For the same dataset, the nodal support values in BI trees are generally higher than those in ML trees. For the same inference method (BI or ML), different data combinations slightly affected the topology and support values. The P123 trees are markedly more resolved, and have overall higher supports at nodes than the others. The ingroup topologies between BI and ML trees are identical based on the P123 and P123R datasets, but are inconsistent based on the P12 and P12R datasets, indicating that the inclusion of the third codon positions make topologies more stable in both ML and BI trees. The nodal supports of phylogenetic trees based on P123 dataset are higher than that of P123R dataset. A similar situation was observed between P12 and P12R trees, reflecting that the exclusion of the rRNA genes can improve branch supports of phylogenetic trees. The monophyly of the infraorder Gryllidea was well supported by all datasets with high nodal supports (PPs = 1; BSs = 100), and consistent with the results proposed by Chintauan-Marquier et al. [17], Zhou et al. [48], Chang et al. [10], Song et al. [8] and Sanno et al. [18].

The monophyletic Grylloidea was confirmed and the relationships within this superfamily were present as Mogoplistidae + (Trigonidiidae + (Phalangopsidae + Gryllidae)). This finding corroborates the generally accepted classification schemes [15] as well as mostly recent studies [8,17,18,39,40,76]. The monophyly of the superfamily Gryllotalpoidea, however, was rejected in the present study. Gryllotalpidae formed the sister taxon to the clade of Myrmecophilidae + Grylloidea rather than solely to Myrmecophilidae. This result is similar to the mitogenome-based trees [8,18], but conflicts with the multilocus-based phylogeny proposed by Chintauan-Marquier et al. [17], which is adopted prevalently as a reference classification. Mitogenomes may experience selective pressures in some insects with peculiar ecological and morphological traits [77,78]. The small and wingless crickets in Myrmecophilidae inhabit subterranean ant nests of low oxygen levels [79,80], whereas mole crickets have larger sizes and short wings, and usually hide in horizontal burrows near the soil surface [81]. The positively selective sites associated with hypoxic adaptability were identified in the *cox1* genes of Myrmecophilidae, but were failed to be detected in those of Gryllotalpidae [18], suggesting that the mitogenomes of Myrmecophilidae and Gryllotalpidae have different evolutionary properties. Therefore, we speculated that the contradictions between mitogenomic and multilocus trees are partially attributed to the evolutionary differences of mitogenomes of the two families. In addition, the inconsistent trees may also be influenced by the lack of nuclear genes, which are important for reconstructing deep-level phylogenetic relationships [82,83,84]. The present investigation improved the resolution of the phylogram by Sanno et al. [18], although more species and markers are necessary for future studies.

In Gryllotalpidae, *G. henana* first split from the remaining gryllotalpids (BSs = 100, PPs = 1) (Figure 6; Supplementary Figures S2–S4). Interestingly, in the second clade, the two specimens of *G. orientalis* were failed to be clustered in one branch. The Korean *G. orientalis* was clustered with the clade of *Gryllotalpa* sp. + *G*. *unispina* based on P123 and P123R datasets (Figure 6, Supplementary Figure S2), but was placed with the clade of the Chinese *G. orientalis* + G. *pluvialis* based on P12 and P12R datasets (Supplementary Figures S3 and S4). Moreover, the K2P genetic distance of the two specimens of *G. orientalis* (0.145) is relatively high compared with the interspecific distances of *Gryllotalpa* (0.022–0.321) (Supplementary Table S7). We speculate that the so-called *G. orientalis* in China is likely a new species, and further morphological and biological evidences are needed to confirm this inference.

## Figures and Tables

**Table 2:** Nucleotide composition of the mitogenomes of six species in Gryllotalpidae.

Feature	Size (bp)	A%	G%	AT%	AT-Skew	GC-Skew
Whole genome	15,504/15,497/15,521/ 15,525/15,506/15,513	35.6/37.2/36.8/ 37.6/37.2/37.1	9.2/10.0/10.3/ 9.6/10.2/10.3	66.4/71.3/70.5/ 72.2/71.0/70.9	0.072/0.043/0.044/ 0.042/0.048/0.047	−0.451/−0.303/−0.302/ −0.309/−0.297/−0.295
PCGs	11,097/11,109/11,091/ 11,064/11,064/11,109	26.5/29.2/29.1/ 29.9/29.0/29.0	16.2/14.4/14.7/ 14.0/14.7/14.7	64.4/70.0/69.4/ 71.1/69.7/69.5	−0.177/−0.166/−0.161/ −0.159/−0.168/−0.165	−0.087/−0.040/−0.039/ −0.031/−0.033/−0.036
1st codon position	3699/3703/3697/ 3688/3688/3703	29.6/30.8/30.4/ 30.6/30.2/30.3	21.7/20.5/20.7/ 20.6/20.7/20.7	61.0/64.5/64.3/ 65.1/63.9/63.8	−0.030/−0.045/−0.054/ −0.060/−0.055/−0.050	0.113/0.155/0.163/ 0.181/0.147/0.144
2st codon position	3699/3703/3697/ 3688/3688/3703	18.4/18.6/18.9/ 19.0/18.8/18.7	15.1/15.4/15.1/ 15.2/15.2/15.2	64.1/64.6/64.9/ 64.9/64.7/64.7	−0.426/−0.424/−0.418/ −0.414/−0.419/−0.422	−0.159/−0.130/−0.140/ −0.136/−0.139/−0.139
3st codon position	3699/3703/3697/ 3688/3688/3703	31.4/38.3/38.1/ 40.3/37.9/38.1	12.0/7.2/8.2/ 6.2/8.2/8.1	68.1/81.0/79.2/ 83.6/80.3/80.2	−0.078/−0.054/−0.038/ −0.036/−0.056/−0.050	−0.248/−0.238/−0.212/ −0.248/−0.168/−0.182
tRNAs	1443/1440/1447/ 1447/1443/1443	37.7/37.8/37.5/ 38.1/37.9/38.0	15.9/15.1/15.0/ 14.7/14.4/14.6	72.3/73.6/73.9/ 74.1/74.4/74.2	0.043/0.027/0.015/ 0.028/0.019/0.024	0.148/0.148/0.149/ 0.140/0.125/0.132
rRNAs	1947/1966/1966/ 2019/2013/1970	31.5/33.7/32.5/ 33.3/32.2/32.2	24.3/18.4/19.2/ 17.7/18.8/19.0	67.1/73.2/71.6/ 73.8/72.9/72.6	−0.061/−0.079/−0.092/ −0.098/−0.117/−0.113	0.482/0.373/0.347/ 0.351/0.387/0.387
CR	863/913/920/ 867/868/917	42.5/40.1/38.2/ 39.5/38.7/39.3	5.2/9.8/10.8/ 10.0/10.4/10.5	81.1/77.3/74.9/ 77.7/76.9/76.8	0.048/0.038/0.020/ 0.017/0.007/0.023	−0.450/−0.137/−0.139/ −0.099/−0.100/−0.099

Data are given as *Gryllotalpa henana*/*G. orientalis* CHN/*G. orientalis* KR/*G. pluvialis*/*Gryllotalpa* sp./*G. unispina*. GC-skew = (G − C)/(G + C), AT-skew = (A − T)/(A + T); CHN, China; CR, control region; KR, Korea.

**Table 3:** Mitogenomic organization of six species in Gryllotalpidae.

Gene	Position		Size	IGN	Codon		Direction
	From	To			Start	Stop	
*trnI*	1/1/1/1/1/1/1	66/65/65/65/65/66	66/65/65/ 65/65/66				J/J/J/J/J/J
*trnQ*	64/63/63/63/63/64	132/130/130/ 130/130/131	69/68/68/ 68/68/68	−3/−3/−3/ −3/−3/−3			N/N/N/N/N/N
*trnM*	180/148/153/ 150/153/159	247/216/221/ 218/221/226	68/69/69/ 69/69/68	47/17/22/ 19/22/27			J/J/J/J/J/J
*nad2*	249/218/223/ 232/230/228	1256/1231/1237/ 1237/1240/1239	1008/1014/1015/ 1006/1011/1012	−1/1/1/13/8/1	GTG/GTG/GTG/ ATT/ATT/GTG	TAA/TAA/T/ T/TAA/T	J/J/J/J/J/J
*trnW*	1261/1230/1238/ 1238/1239/1240	1325/1293/1305/ 1303/1303/1304	65/64/68/ 66/65/65	4/−2/-/-/−2/-			J/J/J/J/J/J
*trnC*	1318/1286/1298/ 1296/1296/1297	1379/1346/1359/ 1356/1357/1358	62/61/62/ 61/62/62	−8/−8/−8/ −8/−8/−8			N/N/N/N/N/N
*trnY*	1379/1347/1360/ 1357/1358/1359	1445/1415/1425/ 1425/1426/1427	67/69/66/ 69/69/69	−1/-/-/-/-/-			N/N/N/N/N/N
*cox1*	1447/1419/1427/ 1427/1428/1429	2980/2952/2960/ 2960/2961/2962	1534/1534/1534/ 1534/1534/1534	1/3/1/1/1/1	ATG/ATG/ATG/ ATG/ATG/ATG	T/T/T/T/T/T	J/J/J/J/J/J
*trnL2*	2981/2953/2961/ 2961/2962/2963	3045/3017/3025/ 3025/3025/3026	65/65/65/ 65/64/64	-/-/-/-/-/-			J/J/J/J/J/J
*cox2*	3046/3019/3027/ 3027/3028/3029	3727/3700/3708/ 3708/3709/3710	682/682/682/ 682/682/682	-/1/1/1/2/2	ATG/ATG/ATG/ ATG/ATG/ATG	T/T/T/T/T/T	J/J/J/J/J/J
*trnK*	3728/3701/3709/ 3709/3710/3711	3797/3770/3779/ 3778/3779/3780	70/70/71/ 70/70/70	-/-/-/-/-/-			J/J/J/J/J/J
*trnD*	3797/3770/3779/ 3778/3779/3780	3862/3834/3843/ 3847/3843/3844	66/65/65/ 70/65/65	−1/−1/−1/ −1/−1/−1			J/J/J/J/J/J
*atp8*	3863/3835/3884/ 3857/3844/3845	4018/3990/3999/ 4003/3999/4000	156/156/156/ 147/156/156	-/-/-/9/-/-	ATT/ATT/ATT/ ATA/ATT/ATT	TAA/TAA/TAA/ TAA/TAA/TAA	J/J/J/J/J/J
*atp6*	4012/3984/3993/ 4000/3996/3994	4689/4661/4669/ 4674/4670/4671	678/678/677/ 675/675/678	−7/−7/−7/ −4/−4/−7	ATG/ATG/ATG/ATA/ATA/ATG	TAA/TAA/TA/ TAA/TAA/TAA	J/J/J/J/J/J
*cox3*	4689/4661/4670/ 4674/4670/4671	5472/5444/5453/ 5457/5453/5454	784/784/784/ 784/784/784	−1/−1/-/ −1/−1/−1	ATG/ATG/ATG/ATG/ATG/ATG	T/T/T/T/T/T	J/J/J/J/J/J
*trnG*	5473/5445/5454/ 5458/5454/5455	5535/5507/5517/ 5520/5516/5517	63/63/64/ 63/63/63	-/-/-/-/-/-			J/J/J/J/J/J
*nad3*	5536/5508/5518/ 5530/5517/5518	5887/5859/5869/ 5872/5870/5869	352/352/352/ 343/354/352	-/-/-/9/-/-	ATT/ATT/ATT/ATA/ATT/ATT	T/T/T/T/TAG/T	J/J/J/J/J/J
*trnA*	5888/5860/5870/ 5873/5869/5870	5951/5921/5932/ 5935/5931/5932	64/62/63/ 63/63/63	-/-/-/-/−2/-			J/J/J/J/J/J
*trnR*	5951/5922/5932/ 5935/5931/5932	6013/5983/5993/ 5996/5992/5993	63/62/62/ 62/62/62	−1/-/−1/ −1/−1/−1			J/J/J/J/J/J
*trnN*	6015/5989/5999/ 6002/5994/5995	6080/6052/6062/ 6066/6057/6058	66/64/64/ 65/64/64	1/5/5/5/1/1			J/J/J/J/J/J
*trnS1*	6081/6053/6063/ 6068/6058/6059	6147/6119/6129/ 6132/6124/6125	67/67/67/ 65/67/67	-/-/-/1/-/-			J/J/J/J/J/J
*trnE*	6149/6135/6141/ 6149/6135/6136	6213/6199/6205/ 6213/6199/6200	65/65/65/ 65/65/65	1/15/11/ 16/10/10			J/J/J/J/J/J
*trnF*	6212/6198/6204/ 6212/6198/6199	6276/6262/6268/ 6276/6262/6263	65/65/65/ 65/65/65	−2/−2/−2/ −2/−2/−2			N/N/N/N/N/N
*nad5*	6277/6263/6269/ 6277/6263/6264	7999/7985/7991/ 7999/7985/7986	1723/1723/1723/ 1723/1723/1723	-/-/-/-/-/-	ATG/ATG/ATG/ATG/ATG/ATG	T/T/T/T/T/T	N/N/N/N/N/N
*trnH*	8003/7987/7993/ 8001/7987/7988	8066/8050/8058/ 8064/8050/8051	64/64/66/ 64/64/64	3/1/1/1/1/1			N/N/N/N/N/N
*nad4*	8067/8051/8059/ 8065/8051/8052	9402/9386/9394/ 9400/9386/9387	1336/1336/1336/ 1336/1336/1336	-/-/-/-/-/-	ATG/ATG/ATG/ATG/ATG/ATG	T/T/T/T/T/T	N/N/N/N/N/N
*nad4L*	9396/9380/9388/ 9394/9380/9381	9692/9676/9684/ 9690/9676/9677	297/297/297/ 297/297/297	−7/−7/−7/ −7/−7/−7	ATG/ATG/ATG/ATG/ATG/ATG	TAA/TAA/TAA/ TAA/TAA/TAA	N/N/N/N/N/N
*trnT*	9695/9679/9687/ 9693/9679/9680	9759/9742/9750/ 9756/9743/9744	65/64/64/ 64/65/65	2/2/2/2/2/2			J/J/J/J/J/J
*trnP*	9760/9743/9751/ 9757/9744/9745	9824/9807/9816/ 9821/9808/9809	65/65/66/ 65/65/65	-/-/-/-/-/-			N/N/N/N/N/N
*nad6*	9827/9810/9819/ 9842/9829/9812	10,345/10,322/10,330/ 10,336/10,323/10,324	519/513/512/ 495/495/513	2/2/2/20/20/2	ATC/ATT/ATT/ATA/ATA/ATT	TAA/TAA/TA/ TAA/TAA/TAA	J/J/J/J/J/J
*cytb*	10,345/10,322/10,331/ 10,336/10,323/10,324	11,478/11,455/11,462/ 11,467/11,456/11,455	1134/1134/1132/ 1132/1134/1132	−1/−1/-/ −1/−1/−1	ATG/ATG/ATG/ATG/ATG/ATG	TAA/TAA/T/T/TAA/T	J/J/J/J/J/J
*trnS2*	11,477/11,454/11,463/ 11,468/11,455/11,456	11,540/11,522/11,530/ 11,536/11,523/11,524	64/69/68/ 69/69/69	−2/−2/-/-/−2/-			J/J/J/J/J/J
*nad1*	11,569/11,548/11,565/ 11,569/11,555/11,556	12,504/12,483/12,500/ 12,504/12,490/12,491	936/936/936/ 936/936/936	28/25/34/ 32/31/31	ATG/ATG/ATG/ATG/ATG/ATG	TAG/TAA/TAA/ TAG/TAA/TAA	N/N/N/N/N/N
*trnL1*	12,506/12,485/12,502/ 12,506/12,492/12,493	12,570/12,549/12,566/ 12,570/12,556/12,557	65/65/65/ 65/65/65	1/1/1/1/1/1			N/N/N/N/N/N
*rrnL*	12,554/12,550/12,567/ 12,571/12,557/12,558	13,767/13,785/13,813/ 13,806/13,793/13,802	1214/1236/1247/ 1236/1237/1245	−17/-/-/-/-/-			N/N/N/N/N/N
*trnV*	13,839/13,786/13,814/ 13,807/13,794/13,803	13,907/13,854/13,882/ 13,875/13,862/13,871	69/69/69/ 69/69/69	71/-/-/-/-/-			N/N/N/N/N/N
*rrnS*	13,909/13,855/13,883/ 13,876/13,863/13,872	14,641/14,584/14,601/ 14,658/14,638/14,596	733/730/719/ 783/776/725	1/-/-/-/-/-			N/N/N/N/N/N
CR	14,642/14,585/14,602/ 14,659/14,639/14,597	15,504/15,497/15,521/ 15,525/15,506/15,513	863/913/920/ 867/868/917				

Data are given as *Gryllotalpa henana*/*G. orientalis* CHN/*G. orientalis* KR/*G. pluvialis*/*Gryllotalpa* sp./*G. unispina*. CHN, China; CR, control region; IGN, intergenic nucleotides; KR, Korea. Negative numbers indicate the overlaps of adjacent genes.

## Data Availability

The data supporting the findings of this study are openly available in National Center for Biotechnology Information (https://www.ncbi.nlm.nih.gov, accessed on 12 April 2022), accession numbers are ON243749 and ON210982.

## Supplementary Materials

The following supporting information can be downloaded at: https://www.mdpi.com/article/10.3390/insects13100919/s1, Table S1: The best partitioning schemes and models for the Bayesian inference (BI) method in different datasets selected by PartitionFinder. Table S2: The best partitioning schemes and models for the Maximum likelihood (ML) method in different datasets selected by ModelFinder. Table S3: The count and relative synonymous codon usage (RSCU) of six species in Gryllotalpidae. Table S4: The numbers of mismatched base pairs in the secondary structure of the tRNAs in the six species of Gryllotalpidae. Table S5: Tandem repeat regions in the control region of six species in Gryllotalpidae. Table S6: Nucleotide diversity (Pi), non-synonymous substitutions rates (Ka), synonymous substitutions rates (Ks), Ka/Ks ratio and genetic distance of six species in Gryllotalpidae. Table S7: The K2P genetic distances in Gryllotalpidae. Figure S1: Location and structure of the potential stem-loops in Gryllotalpidae. (A) The location of the predicted stem-loops in the mitogenome. (B) The structures of potential stem-loops. Figure S2: Phylogenetic tree produced by Bayesian inference (BI) based on the dataset of P123R. Bayesian posterior probabilities (PPs) and bootstrap support values (BSs) are present at nodes. Figure S3: Phylogenetic tree produced by Bayesian inference (BI) based on the P12 dataset. Numerals at nodes are Bayesian posterior probabilities (PPs) and bootstrap support values (BSs). The divergent dotted lines showed the topological tree based on Maximum likelihood (ML) method with underlined bootstrap support values at nodes. Figure S4: Phylogenetic tree produced by Bayesian inference (BI) based on the P12R dataset. Numerals at nodes are Bayesian posterior probabilities (PPs) and bootstrap support values (BSs). The divergent dotted lines showed the topological tree based on Maximum likelihood (ML) method with underlined bootstrap support values.
