# Supplementary material for: Mitogenomic Comparison of the Mole Crickets Gryllotalpidae with the Phylogenetic Implications (Orthoptera: Ensifera)
Source: Insects. 2022 Oct 11;13(10):919. doi: 10.3390/insects13100919 (PMC9604337; doi:10.3390/insects13100919)
Supplement: Supplementary file 1 [file insects-13-00919-s001.zip › insects-1927130-Supplementary Figures.pdf]

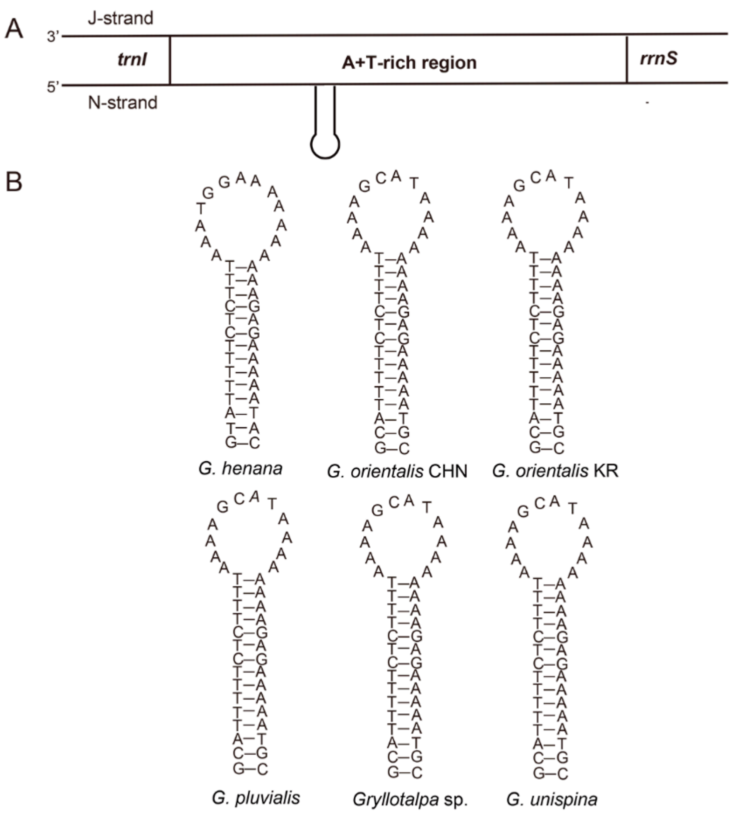


**Figure S1.** Location and structure of the potential stem-loops in Gryllotalpidae. **(A)** The location of the predicted stem-loops in the mitogenome. **(B)** The structures of potential stem-loops.

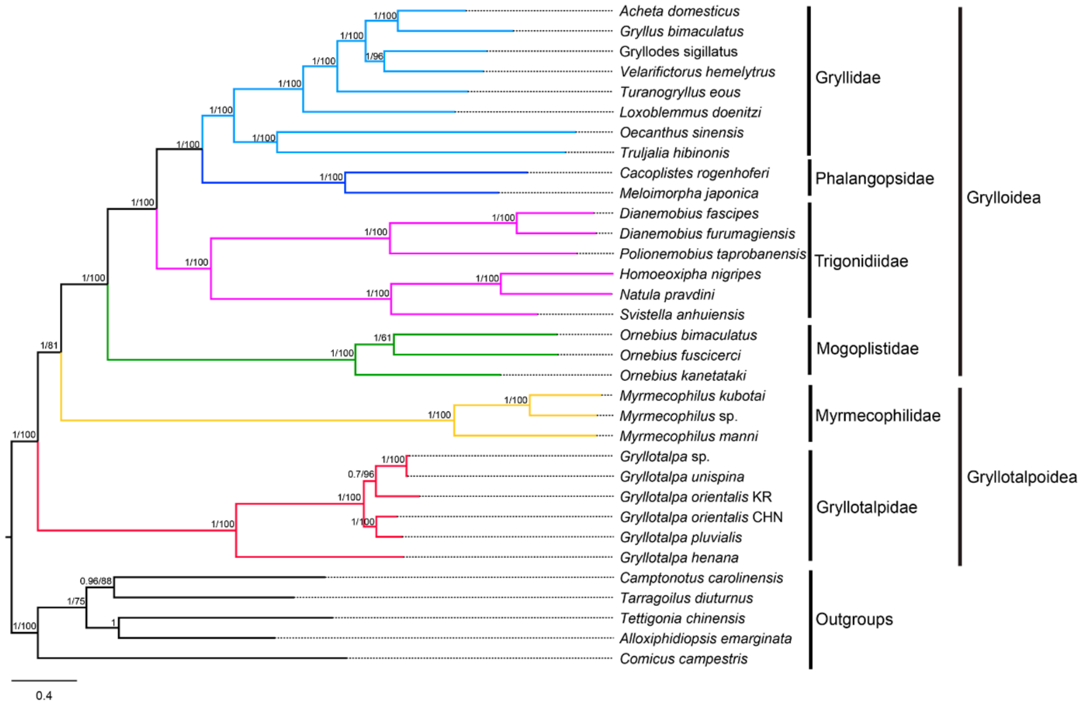


**Figure S2.** Phylogenetic tree produced by Bayesian inference (BI) based on the dataset of P123R. Bayesian posterior probabilities (PPs) and bootstrap support values (BSs) are present at nodes.

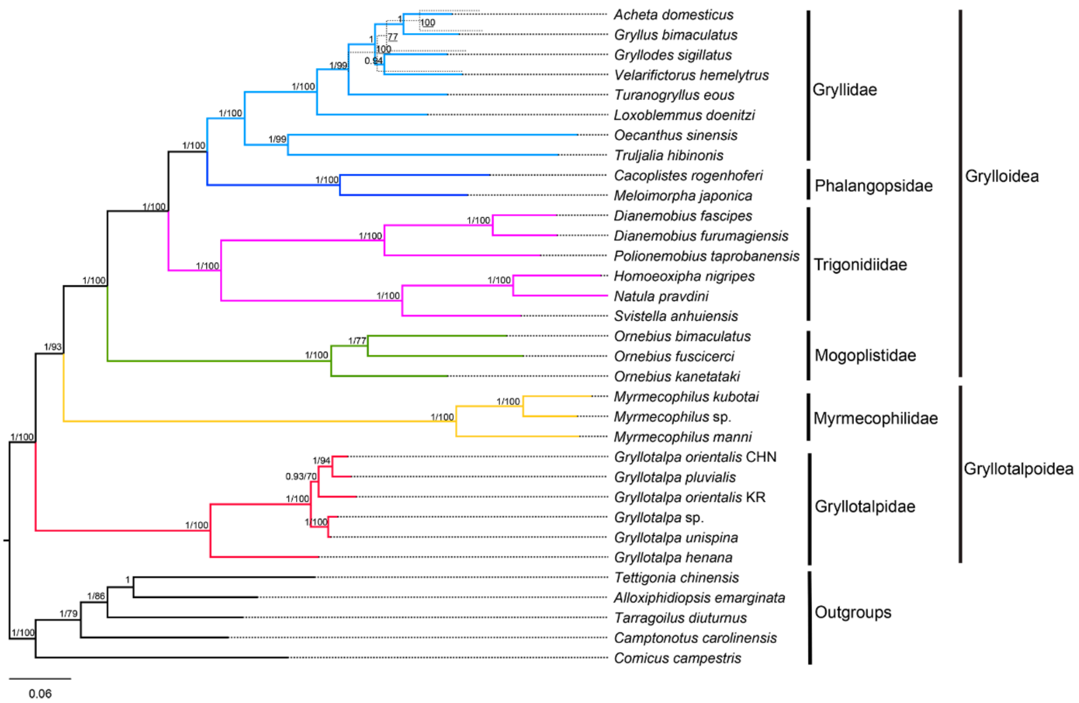


**Figure S3.** Phylogenetic tree produced by Bayesian inference (BI) based on the P12 dataset. Numerals at nodes are Bayesian posterior probabilities (PPs) and bootstrap support values (BSs). The divergent dotted lines showed the topological tree based on Maximum likelihood (ML) method with underlined bootstrap support values at nodes.

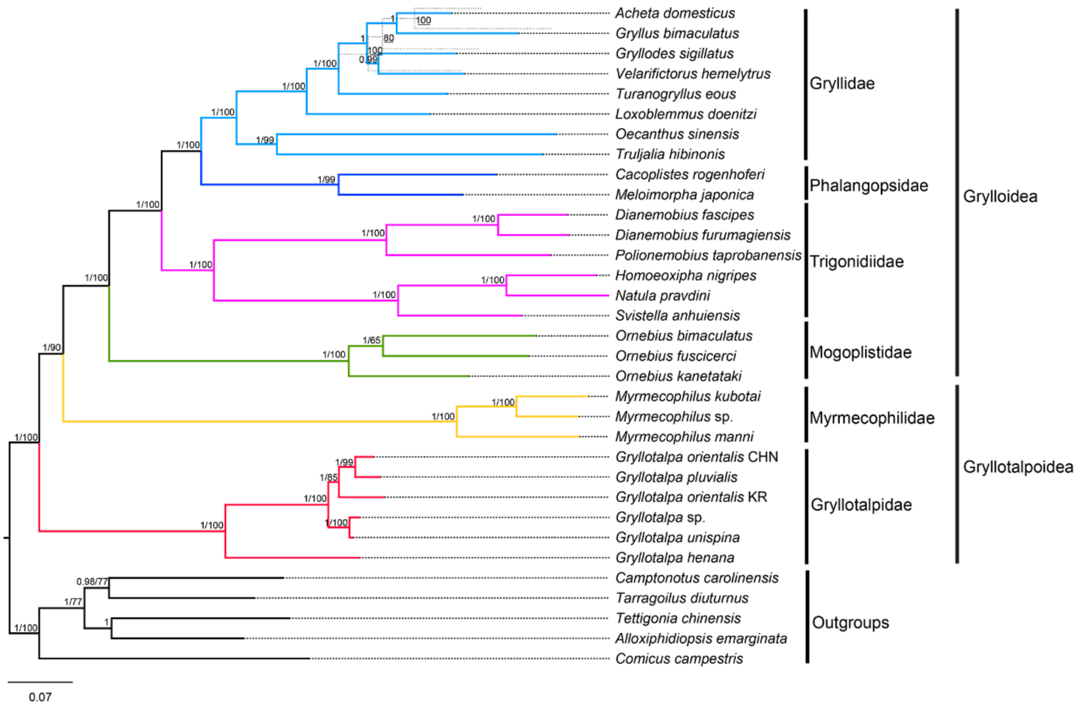


**Figure S4.** Phylogenetic tree produced by Bayesian inference (BI) based on the P12R dataset. Numerals at nodes are Bayesian posterior probabilities (PPs) and bootstrap support values (BSs). The divergent dotted lines showed the topological tree based on Maximum likelihood (ML) method with underlined bootstrap support values.
